# Safety, tolerability and immunogenicity of PRV-101, a multivalent vaccine targeting coxsackie B viruses (CVBs) associated with type 1 diabetes: a double-blind randomised placebo-controlled Phase I trial

**DOI:** 10.1007/s00125-024-06092-w

**Published:** 2024-02-19

**Authors:** Heikki Hyöty, Susanna Kääriäinen, Jutta E. Laiho, Gail M. Comer, Wei Tian, Taina Härkönen, Jussi P. Lehtonen, Sami Oikarinen, Leena Puustinen, Michele Snyder, Francisco León, Mika Scheinin, Mikael Knip, Miguel Sanjuan

**Affiliations:** 1 Department of Virology, Faculty of Medicine and Health Technology, Tampere University, Tampere, Finland; 2 Fimlab Laboratories, Tampere, Finland; 3 Department of Pediatrics, Tampere University Hospital, Tampere, Finland; 4 Clinical Research Services Turku – CRST Oy, Turku, Finland; 5 Provention Bio, Inc., a Sanofi Company, Bridgewater, NJ USA; 6 Research Program for Clinical and Molecular Metabolism, Faculty of Medicine, University of Helsinki, Helsinki, Finland; 7 Institute of Biomedicine, University of Turku, Turku, Finland

**Keywords:** Clinical trial, Coxsackievirus B, Enterovirus, Prevention, Type 1 diabetes, Vaccine

## Abstract

**Aims/hypothesis:**

Infection with coxsackie B viruses (CVBs) can cause diseases ranging from mild common cold-type symptoms to severe life-threatening conditions. CVB infections are considered to be prime candidates for environmental triggers of type 1 diabetes. This, together with the significant disease burden of acute CVB infections and their association with chronic diseases other than diabetes, has prompted the development of human CVB vaccines. The current study evaluated the safety and immunogenicity of the first human vaccine designed against CVBs associated with type 1 diabetes in a double-blind randomised placebo-controlled Phase I trial.

**Methods:**

The main eligibility criteria for participants were good general health, age between 18 and 45 years, provision of written informed consent and willingness to comply with all trial procedures. Treatment allocation (PRV-101 or placebo) was based on a computer-generated randomisation schedule and people assessing the outcomes were masked to group assignment. In total, 32 participants (17 men, 15 women) aged 18–44 years were randomised to receive a low (*n*=12) or high (*n*=12) dose of a multivalent, formalin-inactivated vaccine including CVB serotypes 1–5 (PRV-101), or placebo (*n*=8), given by intramuscular injections at weeks 0, 4 and 8 at a single study site in Finland. The participants were followed for another 24 weeks. Safety and tolerability were the primary endpoints. Anti-CVB IgG and virus-neutralising titres were analysed using an ELISA and neutralising plaque reduction assays, respectively.

**Results:**

Among the 32 participants (low dose, *n*=12; high dose, *n*=12; placebo, *n*=8) no serious adverse events or adverse events leading to study treatment discontinuation were observed. Treatment-emergent adverse events considered to be related to the study drug occurred in 37.5% of the participants in the placebo group and 62.5% in the PRV-101 group (injection site pain, headache, injection site discomfort and injection site pruritus being most common). PRV-101 induced dose-dependent neutralising antibody responses against all five CVB serotypes included in the vaccine in both the high- and low-dose groups. Protective titres ≥8 against all five serotypes were seen in >90% of participants over the entire follow-up period.

**Conclusions/interpretation:**

The results indicate that the tested multivalent CVB vaccine is well tolerated and immunogenic, supporting its further clinical development.

**Trial registration:**

ClinicalTrials.gov NCT04690426.

**Funding:**

This trial was funded by Provention Bio, a Sanofi company.

**Graphical Abstract:**

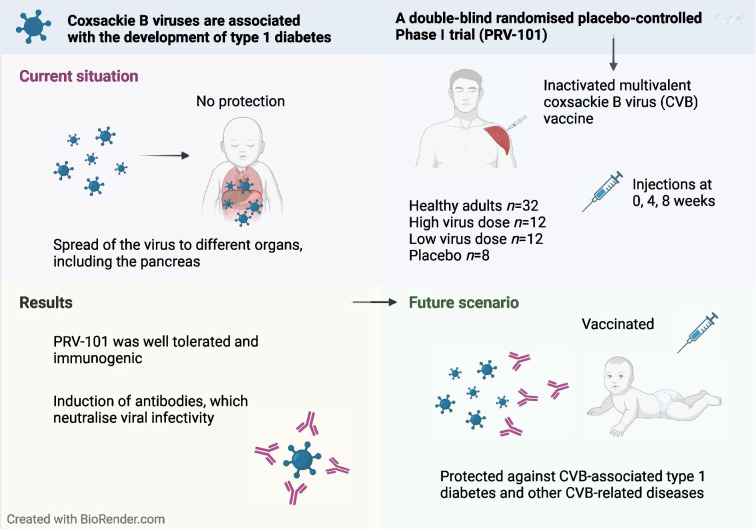

**Supplementary Information:**

The online version contains peer-reviewed but unedited supplementary material available at 10.1007/s00125-024-06092-w.


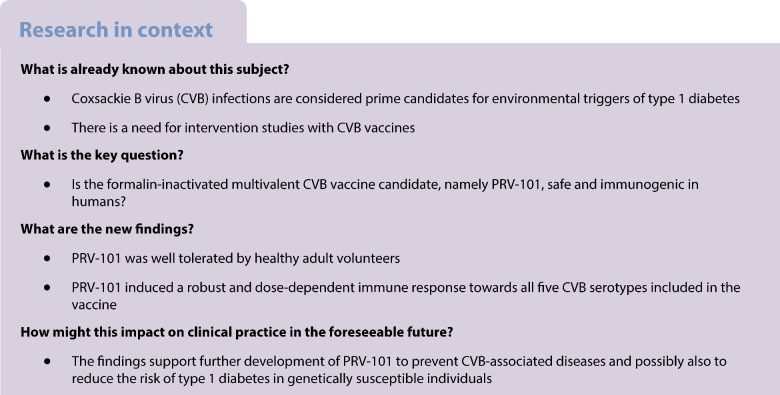


## Introduction

Coxsackie B viruses (CVBs) are common human viruses causing a significant disease burden ranging from mild respiratory symptoms to more severe diseases including myocarditis, pericarditis, meningitis, encephalitis, herpangina, pancreatitis and severe multiorgan infections in infants [1, 2]. Among the more than 110 human enterovirus types, the six CVB types are among the 15 most frequent serotypes reported in healthcare patients in the USA [3, 4].

There is a substantial body of evidence associating CVB infections with type 1 diabetes [2, 5–7]. In addition, prospective studies have shown an association between enterovirus infections, most specifically CVB infections, and the initiation of the beta cell-damaging process in genetically susceptible individuals [5]. Immunohistochemical analysis has revealed the presence of enterovirus capsid protein in pancreatic beta cells in the majority of individuals with type 1 diabetes [8]. This has been confirmed by the detection of enterovirus RNA in the pancreases of such individuals [9–13]. Additional evidence connecting CVBs to type 1 diabetes comes from the observation that beta cells strongly express the cell surface coxsackie and adenovirus receptor (CAR) and that certain CAR gene polymorphisms are associated with type 1 diabetes [5]. CVBs are the only enteroviruses known to use this receptor for cell entry, providing one possible mechanism mediating the tropism of CVBs to human pancreatic beta cells [14].

The exact mechanisms by which CVBs could cause beta cell damage are not known. The detection of viral proteins in beta cells suggests that such cells may be directly infected, with cell damage and inflammation and subsequent initiation of islet autoimmunity [8]. On the other hand, molecular mimicry between viral and beta cell proteins may also play a role [15, 16]. CVBs can also cause persistent infections leading to chronic diseases. For example, acute CVB myocarditis can progress to chronic cardiomyopathy, in which persisting low-grade CVB infection of cardiac myocytes is considered to be the underlying mechanism [17]. CVBs cause persistent infection in the pancreas in mouse models [18], and signs of viral persistence have been observed in type 1 diabetes [5, 11, 19]. CVBs have also been linked to certain other autoimmune disorders such as coeliac disease [13, 20–22].

Enterovirus infections, particularly CVB infections, are currently considered to be prime candidates for environmental triggers of type 1 diabetes, and the need for intervention studies with a CVB vaccine is widely acknowledged [23]. Preclinical studies have demonstrated that both CVB vaccines [24, 25] and anti-CVB antibodies [26] are capable of providing protection against experimental CVB infections and the subsequent development of diabetes. There are precedents for effective formalin-inactivated vaccines against another subgroup of enteroviruses, polioviruses, providing a path for the development of novel CVB vaccines. This, together with the significant disease burden of acute CVB infections and their association with chronic diseases other than diabetes, has prompted the development of human CVB vaccines.

Here, we present the results of the first-in-human trial testing the safety and immunogenicity of a multivalent formalin-inactivated CVB vaccine candidate, PRV-101. PRV-101 is being developed for the prevention of acute CVB infections and the CVB-triggered autoimmune damage to pancreatic beta cells that often progresses to type 1 diabetes [5] and damage to intestinal cells potentially leading to coeliac disease [6, 20].

## Methods

All methods were carried out in accordance with CONSORT guidelines [27].

### Study design and participants

The PROVENT trial (PROtocol for coxsackievirus VaccinE in healthy voluNTeers) was the first-in-human study of the multivalent CVB vaccine PRV-101. It was a Phase I, randomised, double-blind, placebo-controlled, multiple dose escalation study with the primary objective of evaluating the safety and tolerability of up to three PRV-101 i.m. injections at two dose levels. The secondary objective was to evaluate the vaccine’s immunogenicity by analysing viral antibody responses. The PROVENT study was carried out among healthy adult volunteers at a single research centre (Clinical Research Services Turku [CRST]) in the city of Turku, in Southwest Finland, in compliance with applicable regulatory requirements, including adherence to Good Clinical Practice and the ethical principles that have their origin in the Declaration of Helsinki. Ethical approval was obtained from the Ethics Committee of the Hospital District of Southwest Finland. A Data Monitoring Committee (DMC) was established for the PROVENT trial and the study was prospectively registered on the ClinicalTrials.gov database (trial registration no. NCT04690426). The trial protocol was approved by the Finnish Medicines Agency (Fimea).

Study participants were recruited by CRST from the general population by placing advertisements on bulletin boards, social media and other websites, and in local newspapers. Individuals registered in CRST’s database for healthy volunteers were also contacted directly by e-mail. Participants were offered monetary compensation for their time.

The main inclusion criteria were good general health, age between 18 and 45 years, provision of written informed consent and willingness to comply with all trial procedures. Individuals with coeliac disease (or coeliac autoantibodies at diagnostic titres), type 1 diabetes (or diabetes-associated autoantibodies at diagnostic titres), history of drug or food allergy, any autoimmune disease or immunodeficiency, any acute illness or activation of a chronic illness, or other issues that may have affected participant safety or the evaluation of the trial assessments by the investigator were excluded (see Eligibility criteria in electronic supplementary material [ESM] Methods).

Written informed consent was obtained from 44 prospective study participants. After the screening evaluations, 32 participants, 17 men and 15 women as self-reported, with an age range of 18–44 years, were found to be eligible for the study and were enrolled into two PRV-101 dosing cohorts (16 in the low-dose cohort [Cohort 1] and 16 in the high-dose cohort [Cohort 2]). To be able to assess the ability of PRV-101 to cause seroconversion to antibody positivity for all five CVB serotypes, the aim was to enrol at least six participants who were negative in neutralisation antibody assays for each of the five CVB serotypes at baseline. To increase the likelihood of achieving this goal, the clinical trial protocol allowed an expansion of the sample size of Cohort 2, based on the baseline antibody results of Cohort 1. Expansion of Cohort 2 was not needed as the number of initial CVB seronegative participants in Cohort 1 turned out to be acceptable in a blinded review performed by the DMC. Seronegative serology to CVB subtypes at baseline was most common for CVB4 (15/16 participants) and least common for CVB3 (10/16 participants) in Cohort 1.

### Randomisation and blinding

Participants were randomised to each of the dosing cohorts at a ratio of 3:1 (PRV-101:placebo) as follows: participants in Cohort 1 (*n*=16) received either a low dose (100 μl) of PRV-101 (*n*=12) or placebo (*n*=4); and participants in Cohort 2 (*n*=16) received either a high dose (500 μl) of PRV-101 (*n*=12) or placebo (*n*=4). The placebo product was identical to the active drug in appearance and composition, apart from the virus particles. Treatment allocation (PRV-101 or placebo) was based on a computer-generated randomisation schedule (see Randomisation, dosing and follow-up in ESM Methods). Unblinded pharmacy staff at the study site had access to the randomisation schedule and assigned and prepared the study drug for each participant. Other clinical study team members were blinded. Unblinded personnel included an unblinded clinical monitor, pharmacovigilance personnel and unblinded statistician.

### HLA genotyping

HLA-DR/DQ genotyping was carried out stepwise, beginning with the definition of HLA-DQB1 alleles using oligonucleotide probes and then expanding to cover informative HLA-DQA1 alleles known to exist in haplotypes with HLA-DQB1 alleles. In HLA-DQB1*03:02-positive samples, various HLA-DRB1*04 subtypes were also determined. The procedure and methods have been described in detail elsewhere [28]. The method is relevant for distinguishing haplotypes associated with susceptibility to or protection against type 1 diabetes or coeliac disease [29]. HLA genotyping was performed by Tykslab (Turku, Finland) as the service provider.

### Materials and procedures

The PRV-101 product is a sterile solution of a multivalent CVB vaccine, composed of five formalin-inactivated CVB serotypes (CVB 1–5). CVB6 was not included due to its rarity and lack of reported association with type 1 diabetes. An extractable volume of 0.5 ml of PRV-101 was supplied in glass vials. The drug substance was manufactured by Provention Bio, in collaboration with Intravacc (Bilthoven, the Netherlands) in compliance with Good Manufacturing Practices (GMP).

Starting from the randomisation (week 0/day 1) visit, each participant was to receive up to three i.m. injections of the study drug (PRV-101 or placebo) at intervals of 4 weeks in a double-blinded manner. Injections were given into the deltoid muscle of alternating arms, starting from the non-dominant side. After the last dosing visit, each participant was followed for safety for another 24 weeks. An end-of-study (EOS) visit was performed 24 weeks after the third dosing visit (i.e. at week 32). Blood and nasal swab samples were collected at weeks 0, 4, 8, 12 and 32 (see Sample collection in ESM Methods).

### Outcomes

The primary endpoint was the safety and tolerability of PRV-101, assessed by occurrence of treatment-emergent adverse events (TEAEs), including serious adverse events (SAEs), adverse events leading to treatment discontinuation, injection site reactions (frequency and severity), clinically significant changes in vital signs, ECG and safety laboratory test results, type 1 diabetes- or coeliac disease-associated autoantibodies and markers of glucose metabolism (see Autoantibody analyses in ESM Methods). The secondary endpoint was the immunogenicity of PRV-101. This was assessed by analysing neutralising antibodies against each of the CVB strains that were included in the vaccine and by measuring IgG, IgM and IgA class CVB antibodies using ELISA methods (see Virus analyses in ESM Methods). All personnel involved in the analyses remained unaware of the participants’ treatment allocation.

### Statistical analyses

All analyses were performed on the intention-to-treat population, defined as all individuals who were randomised to the study. Data are presented using descriptive statistics. Antibody levels were log-transformed to present the variation in the vaccine-induced antibody responses. Mean and median values of the log-transformed antibody levels were used in representative figures. R version 4.2.1 (www.r-project.org, accessed 23 June 2023) was used for statistical analysis.

## Results

**Fig. 1:**
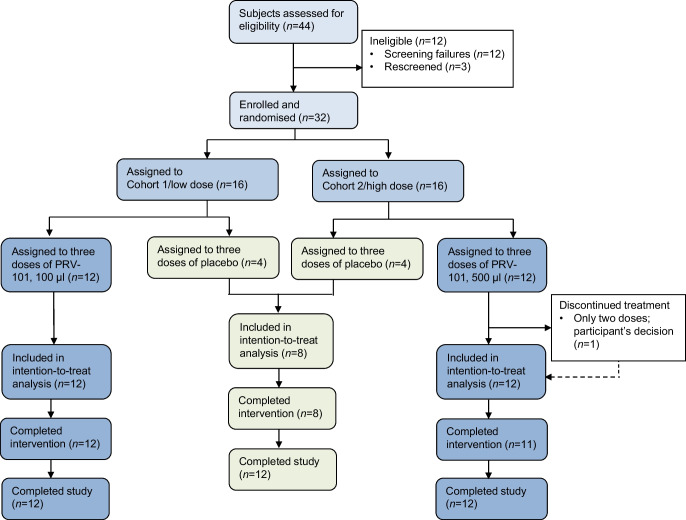
Flow diagram of study participation. There were no dropouts as all participants completed the follow-up. One participant in Cohort 2 did not receive the third injection of PRV-101; all other participants received all three injections of either PRV-101 or placebo

A total of 44 prospective study participants were assessed for eligibility, 32 of whom met all eligibility criteria and were enrolled in the study. These 32 participants were randomised between 14 December 2020 and 10 April 2021 as follows: eight participants received placebo, 12 received the lower dose of PRV-101 in Cohort 1 and 12 received the higher dose of PRV-101 in Cohort 2 (Fig. 1). All study participants completed the study and attended the EOS visit. One participant in Cohort 2 received only two injections of PRV-101 due to family relocation, while the other participants received all three injections according to the study protocol. All 32 participants were assessed for the primary outcome and all scheduled blood and nasal swab samples were obtained from them. Their baseline characteristics are summarised in Table 1. The markers of glucose metabolism (fasting C-peptide and HbA_1c_) were similar in all three groups. Eleven of the study participants carried HLA genotypes conferring high risk for type 1 diabetes and four carried HLA genotypes conferring moderately increased risk, as defined previously [29]. Fifteen participants carried coeliac-disease-associated HLA genotypes [30].

**Table 1:** Demographics and baseline characteristics of the 32 individuals who participated in the study

Characteristic	Placebo	PRV-101, 100 μl	PRV-101, 500 μl	Total
*n*	8	12	12	32
Age, years	28.5 (8.0)	25.8 (7.2)	30.9 (9.2)	28.4 (8.2)
Sex				
Male	5 (63)	9 (75)	3 (25)	17 (53)
Female	3 (38)	3 (25)	9 (75)	15 (47)
Race and ethnicity^a^				
White and non-Hispanic	8 (100)	12 (100)	12 (100)	32 (100)
Weight, kg	72.2 (10.2)	77.9 (11.0)	72.5 (12.5)	74.4 (11.4)
BMI, kg/m^2^	24.3 (2.4)	24.8 (2.6)	24.9 (3.3)	24.7 (2.8)
Fasting C-peptide, nmol/l	0.75 (0.19)	0.56 (0.18)	0.72 (0.24)	0.67 (0.22)
HbA_1c_, mmol/mol	32.3 (1.8)	32.8 (2.0)	34.2 (2.7)	33.2 (2.4)
HbA_1c_, %	5.1	5.2	5.3	5.2

Categorical data are presented as *n* (%) and continuous variables are presented as mean (SD)

^a^Self-reported

There were no SAEs or adverse events leading to study treatment discontinuation. All participants in the placebo group and all but one of the participants in the PRV-101 groups had at least one TEAE. TEAEs occurring in three or more participants in any group are listed in Table 2 by Medical Dictionary for Regulatory Activities (MedDRA) preferred term (https://www.meddra.org/ accessed 20 December 2021). TEAEs comprised all adverse events including those characterised as vaccine reactions and injection site reactions of sufficient severity in the opinion of the investigator. Headache and injection site pain were the most common (Table 2). Based on investigator-reported causality evaluation, those TEAEs that were considered to be related to the study drug (PRV-101 or placebo) occurred in 37.5% of the participants in the placebo group and 62.5% in the PRV-101 group. Among the PRV-101-vaccinated study participants, the most common of these causally evaluated TEAEs were injection site pain, headache, injection site discomfort and injection site pruritus, observed in 33.3%, 20.8%, 16.7% and 12.5% of participants, respectively. One participant in the placebo group reported a severe TEAE of neck pain whereas no severe TEAEs were reported in the PRV-101 groups.

**Table 2:** TEAEs occurring in three or more individuals in any group by MedDRA preferred term

Adverse event	Placebo (*N*=8)	PRV-101, 100 μl (*N*=12)	PRV-101, 500 μl (*N*=12)	PRV-101 total (*N*=24)
Headache	3 (37.5)	7 (58.3)	7 (58.3)	14 (58.3)
Injection site pain	4 (50.0)	4 (33.3)	5 (41.7)	9 (37.5)
Nasopharyngitis	2 (25.0)	1 (8.3)	7 (58.3)	8 (33.3)
Dysmenorrhoea	1 (12.5)	1 (8.3)	3 (25.0)	4 (16.7)
Influenza-like illness	0 (0.0)	1 (8.3)	3 (25.0)	4 (16.7)
Injection site discomfort	1 (12.5)	0 (0.0)	4 (33.3)	4 (16.7)
Injection site pruritus	0 (0.0)	1 (8.3)	2 (16.7)	3 (12.5)
Myalgia	1 (12.5)	1 (8.3)	2 (16.7)	3 (12.5)
Neck pain	4 (50.0)	2 (16.7)	1 (8.3)	3 (12.5)
Seasonal allergy	0 (0.0)	0 (0.0)	3 (25.0)	3 (12.5)

Data are presented as *n* (%)

The table shows the TEAEs occurring in three or more participants in any group by Medical Dictionary for Regulatory Activities (MedDRA) preferred term, Safety Population (https://www.meddra.org/; accessed 20 December 2021). TEAEs included any adverse event, vaccine reaction or injection site reactions of sufficient severity to be considered an adverse event

Local injection site reactions were reported separately, in three (37.5%) participants receiving placebo and in ten (41.7%) participants receiving PRV-101. These included injection site pain, discomfort, pruritus, paraesthesia and/or erythema. No apparent trends were observed in the safety laboratory values. Markers of glucose homeostasis remained within the normal range in all study participants. One participant in the high-dose PRV-101 group showed IAA seroconversion from negative at baseline to low positive at week 12, and remained IAA-positive at week 32. However, this participant was IAA negative in all samples when tested at a speciality reference laboratory using a similar radiobinding assay (RBA) as well as with an electrochemiluminescence (ECL)-based assay for IAA. None of the trial participants experienced seroconversion to positivity for coeliac disease-associated autoantibodies.

**Fig. 2:**
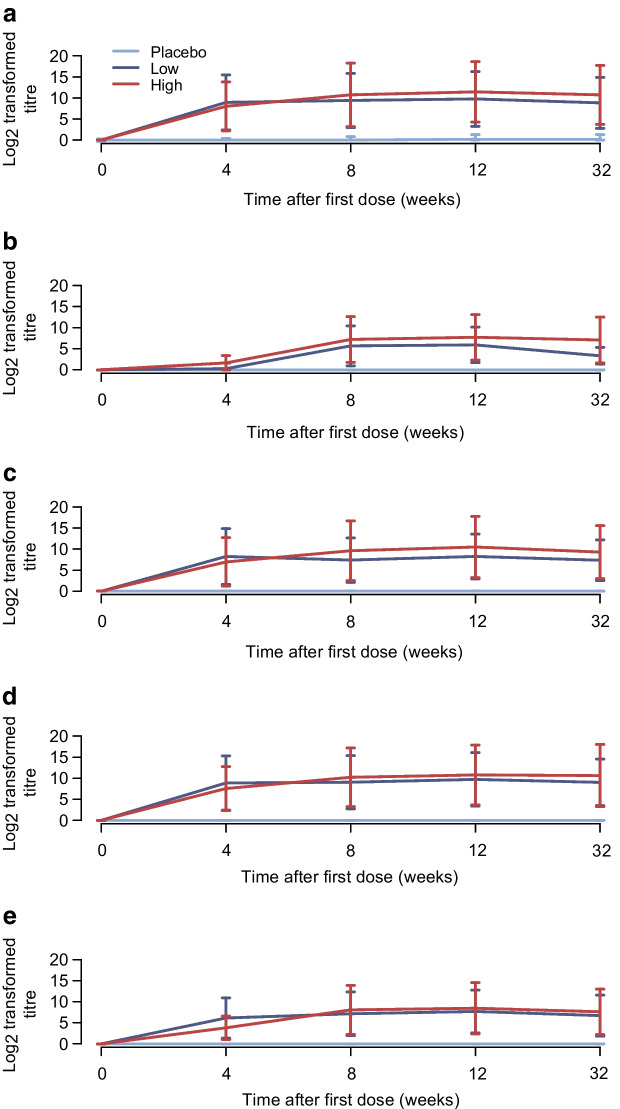
Logarithmic mean titres (±SD) of neutralising antibodies (*y*-axis) against each CVB type at consecutive visits in participants testing negative for the corresponding CVB antibodies at baseline (week 0): (**a**) CVB1; (**b**) CVB2; (**c**) CVB3; (**d**) CVB4; (**e**) CVB5. Three PRV-101 injections were administered at weeks 0, 4 and 8, respectively. The respective numbers of initially seronegative participants in the placebo, low-dose and high-dose groups were as follows: CVB1 7, 11, 11; CVB2 6, 10, 11; CVB3 6, 7, 10; CVB4 7, 11, 8; and CVB5 4, 9, 4

PRV-101 is primarily intended to induce protective immunity in CVB seronegative individuals. Therefore, data from the individuals negative for CVB antibodies at baseline are the most relevant for this goal. Among the participants seronegative at baseline, a clear dose–response relationship was observed in neutralising CVB antibody titres, with higher antibody titres being observed in the high-dose group than in the low-dose group (Fig. 2). Both high- and low-dose PRV-101 groups showed increasing neutralising antibody titres during the vaccination period for all five CVB serotypes. The magnitudes of the responses were different for the five different CVB serotypes but all (100%) of the initially seronegative participants turned seropositive at least at one time point during the study in both PRV-101 groups (ESM Fig. 1). In the low-dose PRV-101 group, 100%, 75%, 100%, 100% and 100% of the initially seronegative participants developed the presumably protective titre ≥8 for CVB1, CVB2, CVB3, CVB4 and CVB5, respectively. In the high-dose group, all participants developed titres ≥8 for CVB1–5. The peak antibody titres showed dose proportionality for each of the five CVB serotypes (ESM Table 1). Seven of the 32 participants were initially seronegative for all five CVB serotypes at baseline. Three of four such participants in the low-dose PRV-101 group (75%) and all such participants in the high-dose group (*n*=3) developed protective neutralising titres (titre ≥8) against all five CVB serotypes, a response that was still seen at the EOS/week 32 visit. In the placebo group, the participants initially classified as seronegative remained seronegative throughout the study, except for one participant who developed low levels of antibodies (titre of 8) against CVB1 (ESM Fig. 1). This participant had low CVB1 antibody titres already in the screening visit sample, suggesting that this apparent seroconversion might have reflected assay variation or natural fluctuation of low antibody titres during the trial from a titre of 4 at the screening visit to titres 2, 4, 6, 8 and 8 at the later visits. None of the study participants was positive for enterovirus RNA in nasal swab samples.

When data from all participants (those initially seropositive and seronegative) were analysed as one group, all 12 participants in the high-dose group and eight (67%) of the 12 participants in the low-dose group met the definition of a responder for each of the five serotypes (seroconversion in initially seronegative participants or a fourfold or greater increase in neutralising antibody titres in initially seropositive participants at any time during the study) (ESM Table 2). The median peak titres against the five CVB serotypes ranged from 30 to 768 in the low-dose group and from 128 to 2048 in the high-dose group. Peak titres were usually reached after the third dose, at or before the week 12 visit, similarly in both PRV-101 groups. Among those participants who had been seropositive at baseline, the majority reached peak titres already after the first dose of PRV-101, suggesting that the vaccine served as a booster of the pre-existing immunity.

Antibodies against CVBs belonging to classes IgG, IgM and IgA were analysed using an ELISA method that was not serotype specific but assessed the overall immunogenicity of PRV-101. IgG levels increased markedly within 4 weeks after the first PRV-101 dose and remained elevated at the later sampling time points, showing slight decreases towards the end of the study (ESM Figs 2, 3). The magnitude of this response was higher in the high-dose group than in the low-dose group (mean 11.2-fold vs 5.6-fold increase, respectively, when compared with baseline). At the EOS/week 32 visit, antibody levels still remained 5.4-fold and 3.8-fold higher compared with baseline, respectively. IgM responses varied considerably between individuals in both dosing groups. The mean IgM levels peaked within 4 weeks, being 11.4-fold higher when compared with baseline in the high-dose group and 11.0-fold higher in the low-dose group, with levels gradually declining at subsequent time points (ESM Figs 2, 3). PRV-101 also induced IgA class CVB antibodies, peaking at week 4, with a 3.7-fold mean increase in the high-dose group and a 4.1-fold increase in the low-dose group compared with baseline levels, varying considerably between individuals. IgG, IgM and IgA levels remained unchanged in the placebo group. ELISA antibody responses were similar in participants who were negative for neutralising antibodies against all tested CVB serotypes at baseline (*n*=7) and those who had neutralising antibodies against at least one CVB type (*n*=17) (ESM Fig. 4).

## Discussion

PRV-101 met the primary safety endpoint of the PROVENT trial, providing initial evidence of acceptable safety and tolerability of the product in healthy adult volunteers. No treatment-emergent SAEs, or adverse events leading to study drug discontinuation or study withdrawal were observed. All TEAEs in the PRV-101 groups were mild to moderate in severity. In addition, the vaccine-induced robust antibody responses to all five CVB serotypes included in the product. Altogether, these findings create a solid basis for the future development of the PRV-101 vaccine candidate.

The observation of robust neutralising antibody responses against the five CVB serotypes is highly important for the future development of PRV-101. Neutralising antibodies are specific for individual CVB serotypes and this finding therefore indicates that each inactivated virus type was immunogenic in the vaccine. Of note, previous studies with the poliovirus vaccine have shown that neutralising antibodies mediate protection against the virus [31, 32].

PRV-101 induced a clear increase in neutralising CVB antibody titres in both initially CVB seropositive and seronegative participants. In the initially seronegative participants, a clear dose–response relationship was observed, as the higher PRV-101 dose induced higher antibody levels than the lower dose. The results also showed the durability of the antibody response as all participants in the high-dose group maintained antibody titres of 8 or higher until the end of the study, with the exception of one participant whose CVB2 antibodies decreased to a titre of <4. The cut-off titre 8 corresponds to antibody levels that have been considered protective against another enterovirus, the poliovirus, measured as protection against paralysis as a consequence of blocking the spread of the virus to the central nervous system [33]. The high seroconversion rate among initially seronegative participants (100% across all CVB serotypes in both dosing groups) is another indicator of relevant immunogenicity. The finding that peak neutralising antibody titres occurred after the third vaccination suggests a booster effect of repeated vaccinations.

At the end of the study, 6 months after the third dose of the vaccine, >90% of participants had titres of 8 or higher against all five serotypes. The neutralising antibody levels were comparable with the neutralising poliovirus antibody levels previously seen in children who had received three or four doses of an inactivated poliovirus vaccine a few months before sampling, when using the same plaque reduction assay as in the current study [34]. The magnitude of the PRV-101-induced CVB antibody responses also compares well with those induced by prototype CVB vaccines in our preclinical studies in mice and rhesus macaques [24, 25]. These preclinical studies were carried out using a formalin-inactivated multivalent CVB vaccine similar to PRV-101, and antibodies were analysed using the same plaque reduction assay. The vaccinated animals were efficiently protected against experimental CVB infection.

In the current study, PRV-101 also induced high levels of IgG class CVB antibodies as measured with ELISA. Dose-dependent IgG responses developed rapidly, were already apparent 1 month after the first vaccine injection and were long-lasting, as the IgG levels remained elevated at the end of the study. PRV-101 also induced IgM and, somewhat more variably, IgA class antibody responses but these were less robust than the IgG responses. The IgA responses suggest that PRV-101 may also have the potential to induce mucosal immunity, even if such responses may be weak and vary from one individual to another.

The neutralising antibody response to CVB2 was somewhat weaker than the responses to the other CVB serotypes. This may be due to differences in the proportions of the individual CVB serotype components in the PRV-101 product, as both the protein concentration and the viral particle number of the CVB2 component were lower than those of the other components. In our preclinical animal studies with similarly inactivated CVB vaccines, CVB2 was equally immunogenic as the other CVBs, suggesting that the immunogenicity of CVB2 per se does not markedly differ from the other CVBs. Together with the clear dose–response pattern seen in the high-dose and low-dose PRV-101 groups, these findings suggest that the immunogenicity of each CVB component of PRV-101 may be optimised by adjusting their relative proportions in the vaccine.

PRV-101 was well tolerated in this trial and only relatively mild events, including headache, injection site pain or discomfort and itching were associated with dosing of PRV-101, being in line with the experience from the inactivated poliovirus vaccine. From this point of view, the results from this first-in-human trial support the feasibility of continuing this development programme. Biological proof-of-concept of preventing CVB-induced diabetes by a CVB vaccine has recently been obtained from preclinical mouse studies where the prototype CVB vaccine efficiently protected against experimental CVB infections and against beta cell damage and diabetes that might otherwise occur after a CVB infection [24, 25].

The mechanisms by which CVB infections can cause beta cell damage and clinical diabetes are not fully understood. The prevailing hypothesis is that CVBs infect insulin-producing beta cells, leading to cell damage, local inflammation in pancreatic islets, loss of immune tolerance in a genetically predisposed host and initiation of an autoimmune process. Since PRV-101 is an inactivated vaccine, it cannot cause type 1 diabetes by such a mechanism. However, molecular mimicry between CVBs and host proteins might induce cross-reactive immune responses that could lead to cell damage. In fact, one mimicry epitope has been discovered in the non-structural viral protein 2C and the GAD65 autoantigen expressed in beta cells, and this epitope has been shown to be recognised by the immune system [15, 16]. Importantly, PRV-101 does not contain this epitope since it only consists of structural virus proteins. Nevertheless, some other cross-reactive epitopes may potentially still exist in CVBs, so the trial participants were carefully monitored for signs of type 1 diabetes. None of them developed type 1 diabetes, signs of subclinical beta cell dysfunction or clinically relevant levels of diabetes-associated autoantibodies. One trial participant turned weakly positive for IAA during the study but all follow-up samples from this participant were found to be IAA negative in another laboratory using an RBA similar to that employed in the trial. High-affinity IAAs are predictive of progression to type 1 diabetes. An additional, ECL-based assay for detecting high-affinity IAAs [35, 36] was also negative for this trial participant. The IAA-positive participant also remained negative for all other islet autoantibodies. Altogether, we conclude that this participant had no signs of clinically relevant islet autoimmunity. The low-titre and low-affinity IAAs detected by one laboratory but not confirmed by another laboratory may be considered to lie within the background variation and to have no predictive value for the development of type 1 diabetes. These findings are in line with the safety results from toxicological studies with PRV-101, including a 4 week repeat-dose study in mice (data summary available on request via the corresponding author). In addition, other preclinical studies with similar CVB vaccines have indicated no induction of diabetes or IAA in different mouse models or in rhesus macaques. It is also important to note that PRV-101 did not induce autoantibodies against tissue transglutaminase, which would be predictive for coeliac disease, another autoimmune disease that has been linked to CVB infections. Overall, there was no evidence to suggest that PRV-101 would promote autoimmunity, even in genetically predisposed individuals (almost half of the study participants carried HLA markers predisposing to type 1 diabetes and/or coeliac disease).

This study has some limitations. Evaluation of long-term safety and immunogenicity of PRV-101 is needed, as well as studies of T cell-mediated immune responses to CVB that may help to maintain long-term immunity. Inactivated poliovirus vaccination schedules include later booster vaccinations after the primary vaccination regimen and it is possible that this also holds for PRV-101. Studies evaluating PRV-101-induced T cell responses as well as later-stage CVB antibody levels are currently in progress. Another limitation is that the current study only included adults. Therefore, we cannot exclude the possibility that some of the CVB seronegative individuals had been exposed to CVB in the past and that their antibodies had decreased to undetectable levels by the initiation of the current study. In such cases, the pre-existing memory T cells could have facilitated the immune responses induced by PRV-101. Therefore, subsequent studies in young, exposure-naive children are needed. A critical next step would be to initiate a Phase Ib study of PRV-101 in children, particularly infants, who are the future target population of the vaccine.

In conclusion, this first-in-human, proof-of-mechanism study has demonstrated, for the first time, that a multivalent formalin-inactivated CVB vaccine was both well tolerated and immunogenic in humans. The vaccine induced robust and dose-dependent immune responses, in both male and female participants, towards all five CVB serotypes included in the vaccine. Future studies are planned to progressively increase age and ethnic and regional diversity of the programme. The results of this randomised, placebo-controlled trial support further development of this first-in-class vaccine to prevent CVB infections and several CVB-associated diseases, and potentially also ultimately decrease the global incidence and disease burden of type 1 diabetes and coeliac disease.

### Supplementary Information

Below is the link to the electronic supplementary material.

Supplementary file1 (PDF 328 KB)

## Data Availability

The datasets are available from the corresponding author upon reasonable request.

## Abbreviations

CAR: Coxsackie and adenovirus receptor
CRST: Clinical Research Services Turku
CVB: Coxsackie B virus
DMC: Data Monitoring Committee
EOS: End-of-study
GMP: Good Manufacturing Practices
MedDRA: Medical Dictionary for Regulatory Activities
PROVENT: PROtocol for coxsackievirus VaccinE in healthy voluNTeers
RBA: Radiobinding assay
SAE: Serious adverse event
TEAE: Treatment-emergent adverse event

## Authors’ relationships and activities

WT, MSa, GMC, FL and MSn are employed by Provention Bio, Inc, a Sanofi company, which is developing PRV-101. Provention Bio was involved in the design and management of the trial, data collection and analysis. MK and HH are board members and stock owners of Vactech Oy, a Finnish biotech company that has contributed to the early-stage development of the vaccine and participated in the trial design and laboratory analyses. SK and MSc are employees of CRST Oy, a clinical contract research organisation that was contracted by Provention Bio to perform the study. The authors declare that there are no other relationships or activities that might bias, or be perceived to bias, their work.
