# Supplementary material for: Safety, tolerability and immunogenicity of PRV-101, a multivalent vaccine targeting coxsackie B viruses (CVBs) associated with type 1 diabetes: a double-blind randomised placebo-controlled Phase I trial
Source: Diabetologia. 2024 Feb 19;67(5):811–21. doi: 10.1007/s00125-024-06092-w (PMC10954874; doi:10.1007/s00125-024-06092-w)
Supplement: Supplementary file 1 — Supplementary file1 (PDF 328 KB) [file 125_2024_6092_MOESM1_ESM.pdf]

Electronic Supplemental Material (ESM)

Safety, tolerability and immunogenicity of PRV-101, a multivalent vaccine targeting coxsackie B viruses (CVBs) associated with type 1 diabetes: a double-blind randomised placebo-controlled Phase 1 trial

Hyoty et al.

ESM Methods

Eligibility criteria

Subjects were eligible to be included in the study only if all of the following criteria applied: 1) male or female between 18 and 45 years of age; 2) healthy with no clinically significant abnormalities, as determined by medical history, physical examination, blood chemistry assessments, hematologic assessments, urine analysis, vital signs, and ECG; 3) body weight in the range of 50 to 100 kg and body mass index (BMI) in the range of 19 to 30 kg/m^2; 4) Women of childbearing potential (WOCBP) have a negative serum pregnancy test at screening and a negative urine pregnancy test immediately prior to each study drug administration and agreed to use an acceptable method of highly effective contraception from one month prior to the first dose of study drug until 3 months after the end of the study; 5) Men either had had a vasectomy at least 3 months before the first dose of study drug administration or agreed to use highly effective contraception with expected failure rate <1% and do not donate sperm during the study through the final study visit; 6) agree to abstain from alcohol intake 48 hours before each dose of study drug and 48 hours before all other study visits; 7) agree not to use prescription medications within 14 days prior to the first dose of study drug through the end of the study, unless approved by the Principal Investigator (PI) and the Sponsor’s Medical Lead; 8) agree not to use non-prescription medications within 14 days prior to study drug administration unless approved by the PI and Medical Lead; 9) have signed an informed consent form indicating that he or she understood the purpose of and procedures required for the study and was willing to participate in the study; 10) willing and able to adhere to the study visit schedule and other requirements, prohibitions, and restrictions specified in the protocol. Subjects were excluded from the study if any of the following criteria applied: 1) having or having had a history of any clinically significant medical illness or medical disorder that the principal investigator considered should exclude the subject; 2) had celiac disease or celiac autoantibodies at diagnostic titres; 3) had type 1 diabetes or diabetes-associated autoantibodies at diagnostic titres; 4) had an active acute or chronic infection or diagnosed latent infection; 5) had a serious infection or had been hospitalized or received intravenous antibiotics for an infection during the 3 months prior to screening; 6) had an acute illness, including a common cold, within 7 days prior to study drug administration; 7) had had a major illness or hospitalisation within 1 month prior to study drug administration; 8) had a major or traumatic surgery within 12 weeks of screening; 9) plan to undergo elective surgery from 4 weeks prior to study drug administration through the end of the study; 10)

had positive serology results for human immunodeficiency virus (HIV) antibodies, hepatitis B surface antigen (HBsAg), or hepatitis C virus (HCV) antibodies at screening; 11) had a recent history of alcohol or drug abuse within 6 months of screening; 12) had a positive urine toxicology screen result at screening for substances of abuse; 13) had a positive alcohol breath test at screening; 14) had donated blood within 60 days prior to screening; 15) had known or suspected intolerance or hypersensitivity to vaccines or any components of the formulation used in the PROVENT trial; 16) had a history of drug and/or food allergy or other active allergic disease requiring the constant use of medications or a history of severe allergic reaction, angioedema, or anaphylaxis; 17) had received an experimental antibody or biologic therapy within 6 months or any other experimental therapy or investigational agent within 60 days or five half-lives (whichever was longer) before the first study drug administration; 18) had received any live or inactivated virus vaccines, subunit vaccines, or bacterial vaccinations within 4 weeks prior to screening or was expected to receive any live virus or bacterial vaccinations during the study through 6 weeks after the final dose of the study drug; 19) was unable or unwilling to undergo multiple venipunctures because of poor tolerability or lack of easy access to veins; 20) had participated in a previous cohort in the current study or was concurrently participating in another study using an investigational agent or procedure; 21) had any condition that, in the opinion of the Investigator, would compromise the wellbeing of the subject or the integrity of the study, or prevent the subject from meeting or performing study requirements; 22) employees of the investigator or study centre, with direct involvement in the proposed study or other studies under the direction of that investigator or study centre, as well as family members of the employees or the Investigator, were prohibited from participating in the study.

Randomisation, dosing and follow-up

Two subjects (one subject randomised to PRV-101 and one to placebo) formed a sentinel group in Cohort 1. The sentinel subjects were admitted, received the assigned dose, and remained confined at the Emergency Clinic of Turku University Hospital for 24 hours after dosing. Once the sentinel subjects' safety assessments of Day 8 had been completed, and upon satisfactory review of their safety data by the investigator and the sponsor's medical lead, the remaining Cohort 1 subjects were enrolled, randomised and dosed, with staggered initiation of dosing, with at least 30 min intervals and so that no more than four subjects were dosed on a single day.

Randomisation and dosing of Cohort 2 was initiated once all 16 Cohort 1 subjects had received their second dose and the safety results of their subsequent safety visit (7 days after the second dose) were available and had been reviewed by the Data Monitoring Committee. Cohort 2 was also started with two sentinel subjects (one subject randomised to PRV-101 and one to placebo) hospitalised for 24 hours, followed by the remaining subjects treated as indicated above for Cohort 1.

An end-of-study visit was performed 24 weeks after the third dosing visit, i.e., at Week 32. The subjects were also contacted by telephone at 8 and 12 weeks after their third dose

to enquire about possible adverse events (AEs). The overall duration of study participation for each subject was thus up to 38 weeks, including a screening period of up to 6 weeks and 32 weeks from randomisation to the final follow-up visit.

Sample collection

Blood samples were drawn at prespecified time points (weeks 0, 4, 8, 12 and 32) to assess the immunogenicity of PRV-101, including analysis of antibodies against the five CVB serotypes targeted by PRV-101, and to analyse type 1 diabetes- and celiac disease-associated autoantibodies, fasting blood glucose and C-peptide, and haemoglobin A1c (HbA1c). Nasal swabs were collected at the same time points to quantitatively determine the possible presence of enteroviruses, using real-time reverse transcription quantitative polymerase chain reaction (RT-qPCR). Safety assessments throughout the study included AE assessments, recording of concomitant medications, clinical laboratory tests, vital signs, physical examinations, and electrocardiograms (ECG). Blood samples for HLA genotyping were collected at study start. The HLA genotype and CVB serology results remained blinded until the end of the study.

Autoantibody analyses

Four islet autoantibodies [antibodies to insulin (IAA), truncated GAD65 (GAD96-585), islet antigen 2 (IA-2A) and zinc transporter 8 (ZnT8A)] were analysed in the PEDIA laboratory, University of Helsinki, from serum samples collected at each visit with specific radiobinding assays (RBAs) [1]. Celiac disease-related autoantibodies in serum, specifically, anti-transglutaminase IgA and IgG antibodies, as well as all haematology and clinical chemistry safety parameters were analysed by the accredited clinical laboratory of Turku University Hospital with standard methods.

Virus analyses

Neutralising antibody levels were measured against each of the CVB1-5 serotypes using plaque reduction assays [2] by end-point titration using two-fold step-serial dilution series of each serum sample at the Department of Virology, Faculty of Medicine and Health Technology, Tampere University. Neutralising antibodies are known to be serotype-specific, reflecting the immunisation efficacy of each of the CVB components of the vaccine. A titre equal to or larger than 4 (1:4) was considered positive and a titre equal to or larger than 8 (1:8) was considered protective based on the experience from poliovirus vaccines. IgG, IgM and IgA levels in serum were analysed using commercial ELISA kits [SERION ELISA classic Coxsackievirus IgG (ESR134G), IgM (ESR134M) and IgA (ESR134A); Serion GmbH, Würzburg, Germany] according to the manufacturer's instructions. Real-time RT-qPCR for enterovirus RNA was conducted to identify enterovirus infections in nasal swab samples. This RT-qPCR assay was capable of detecting different enterovirus types, including all CVB strains [3]. In addition, a separate RT-PCR assay was used to amplify regions of the viral genome that corresponded to the exact serotype of the virus [4].

References

1. Knip M, Virtanen SM, Seppä K, et al (2010) Dietary intervention in infancy and later signs of beta-cell autoimmunity. N Engl J Med 363(20):1900–1908. https://doi.org/10.1056/NEJMoa1004809

2. Laitinen OH, Honkanen H, Pakkanen O, et al (2014) Coxsackievirus B1 is associated with induction of β-cell autoimmunity that portends type 1 diabetes. Diabetes 63(2):446–455. https://doi.org/10.2337/db13-0619

3. Oikarinen S, Martiskainen M, Tauriainen S, et al (2011) Enterovirus RNA in blood is linked to the development of type 1 diabetes. Diabetes 60(1):276–279. https://doi.org/10.2337/db10-0186

4. Nix WA, Oberste MS, Pallansch MA (2006) Sensitive, seminested PCR amplification of VP1 sequences for direct identification of all enterovirus serotypes from original clinical specimens. J Clin Microbiol 44(8):2698–2704. https://doi.org/10.1128/JCM.00542-06

ESM Results

ESM Table 1. Peak titres of neutralising antibodies in subjects who were seronegative at baseline per CVB type.

Serotype	Placebo N=8	Cohort 1 (low dose) N=12	Cohort 2 (high dose) N=12
**CVB1,** N seronegative	7	11	11
Mean (SD)	1 (3.0)	884 (651)	3025 (1561)
**CVB2,** N seronegative	6	10	11
Mean (SD)	0 (0)	79 (118)	250 (271)
**CVB3,** N seronegative	6	7	10
Mean (SD)	0 (0)	567 (681)	1510 (1410)
**CVB4,** N seronegative	7	11	8
Mean (SD)	0 (0)	867 (547)	2144 (1499)
**CVB5,** N seronegative	4	9	4
Mean (SD)	0 (0)	206 (160)	368 (438)

Seronegativity was defined as an antibody titre <4. Titres are expressed as geometric means (SD).

ESM Table 2. Number and proportion (%) of responders by CVB serotype based on neutralising antibody levels in all 32 subjects participating in the PROVENT trial.

**CVB Serotype**	**Placebo N=8**	**Cohort 1 (low dose) N=12**	**Cohort 2 (high dose) N=12**
Any CVB serotype	1 (12)	12 (100)	12 (100)
All 5 CVB serotypes	0 (0.0)	8 (67)	12 (100)
CVB1	1 (12)	12 (100)	12 (100)
CVB2	0 (0.0)	12 (100)	12 (100)
CVB3	0 (0.0)	9 (75)	12 (100)
CVB4	0 (0.0)	12 (100)	12 (100)
CVB5	0 (0.0)	11 (92)	12 (100)

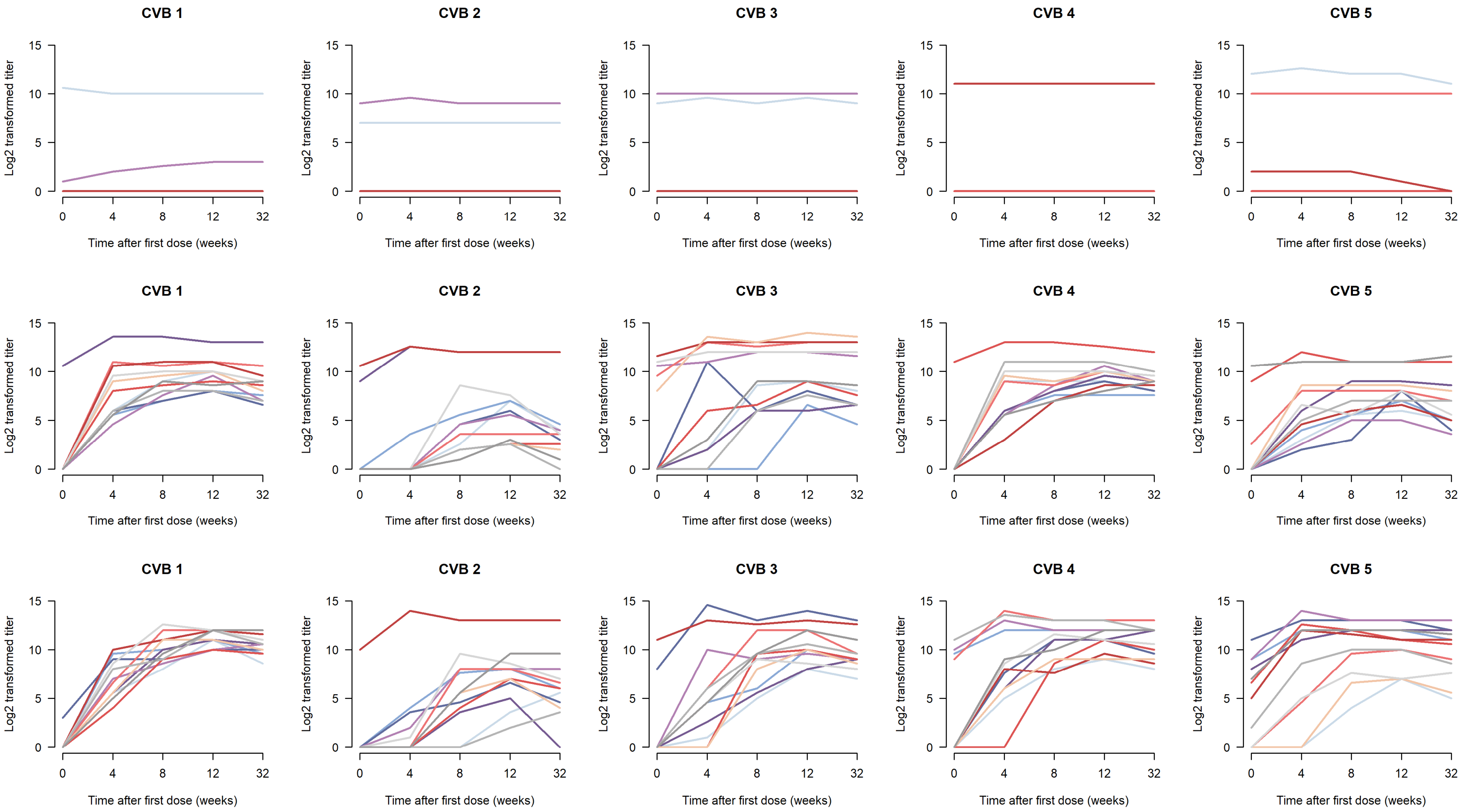

ESM Fig. 1. Individual neutralising antibody responses (log^2 titres of neutralising antibodies; y-axis) against each CVB type at consecutive visits at weeks 0, 4, 8, 12 and 32 (EOS visit) in all 32 participants of the PROVENT trial. Three PRV-101 injections were administered at weeks 0, 4 and 8, respectively. Each subject is marked by a specific color. Top panel=placebo, middle panel=low dose, lower panel=high dose

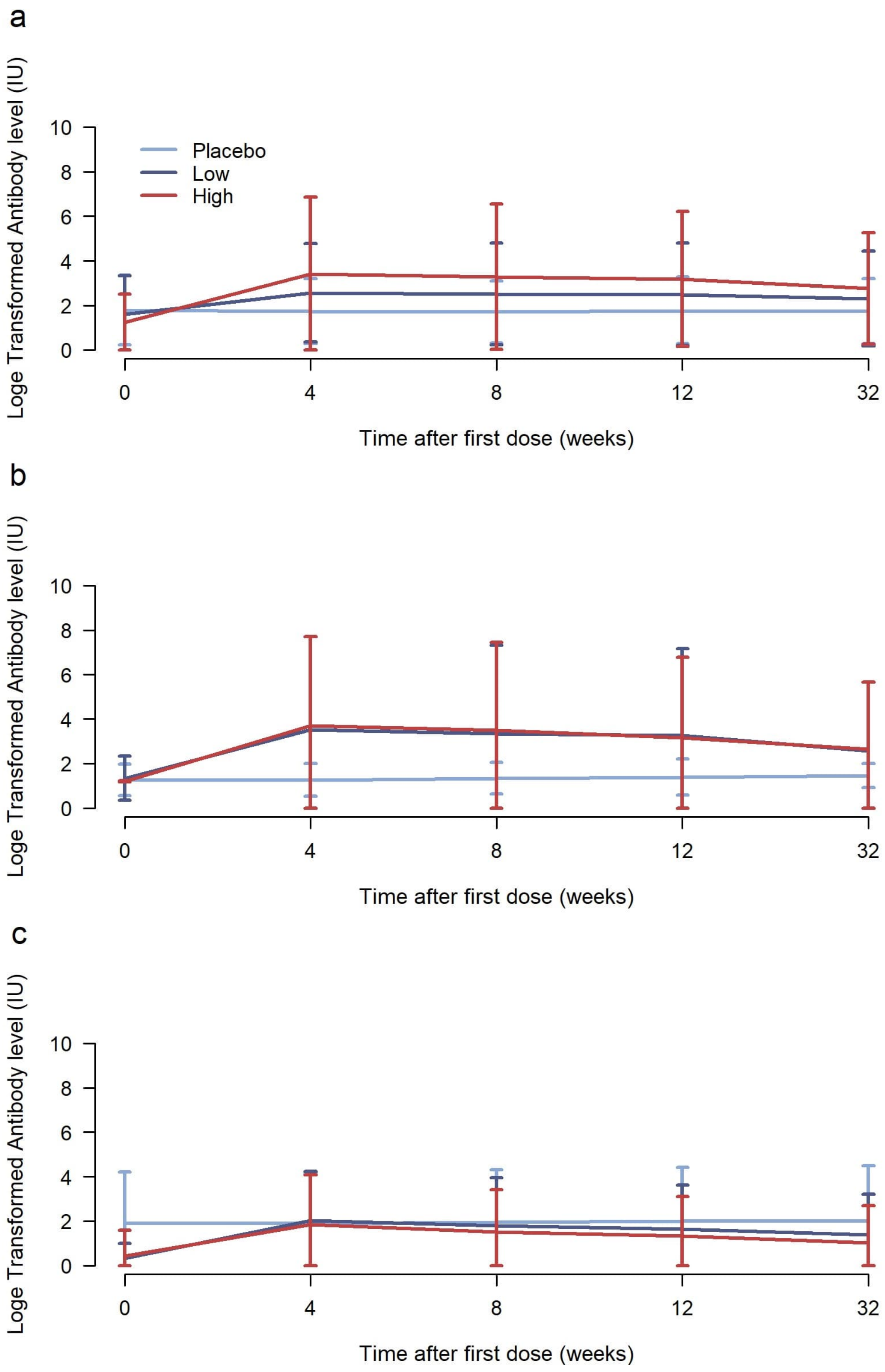

ESM Fig. 2. Logarithmic mean levels (±SD) of IgG (a), IgM (b) and IgA (c) class antibodies against Coxsackie B virus antigen in ELISA at consecutive visits at weeks 0, 4, 8, 12 and 32 (EOS visit) in all 32 subjects participating in the PROVENT trial.

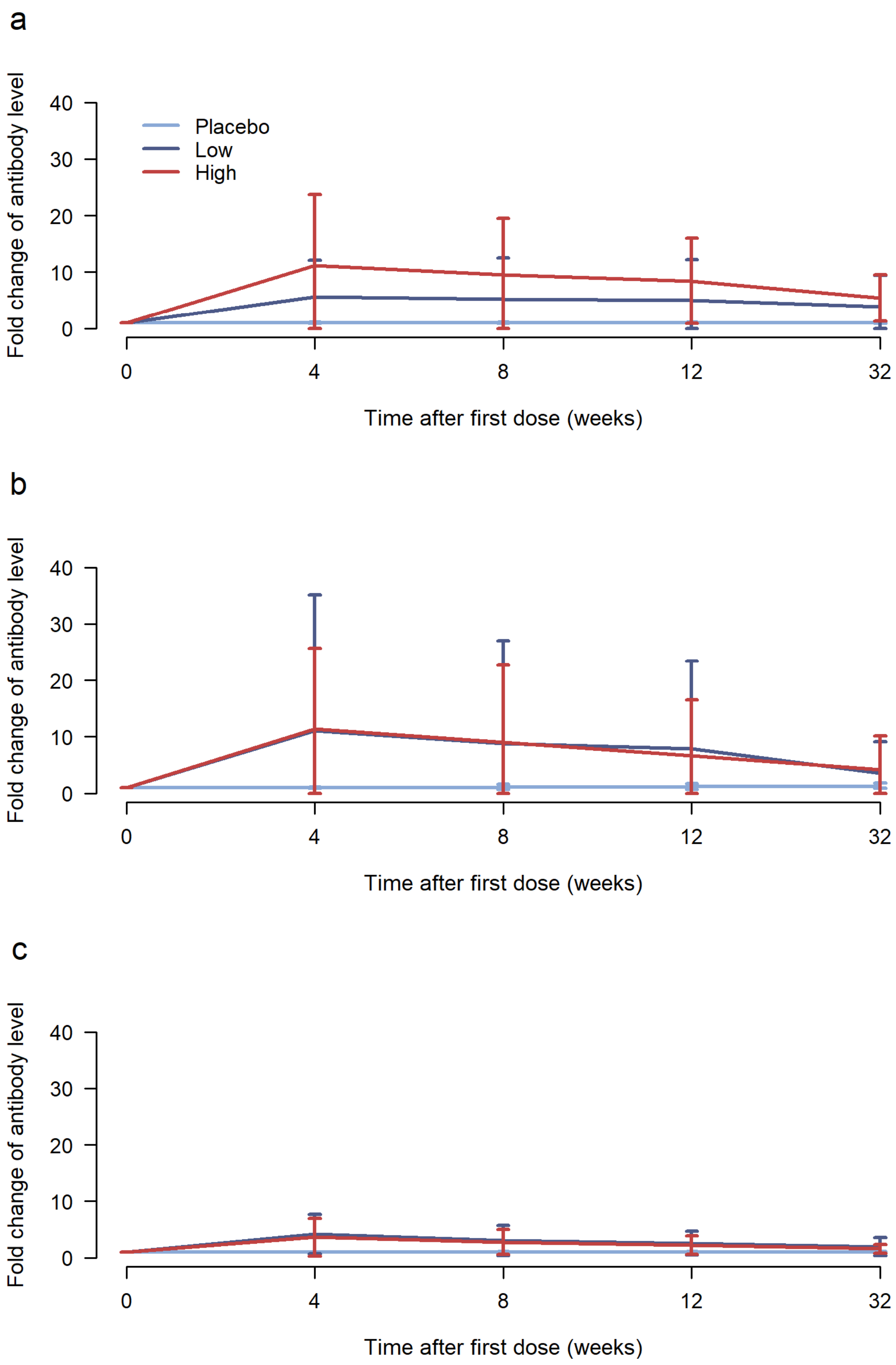

ESM Fig. 3. Mean fold-change (±SD) in IgG (a), IgM (b) and IgA (c) class antibody levels against Coxsackie B virus antigen in ELISA at weeks 4, 8, 12 and 32 (EOS visit) compared to baseline (week 0) in all 32 subjects participating in the PROVENT trial.

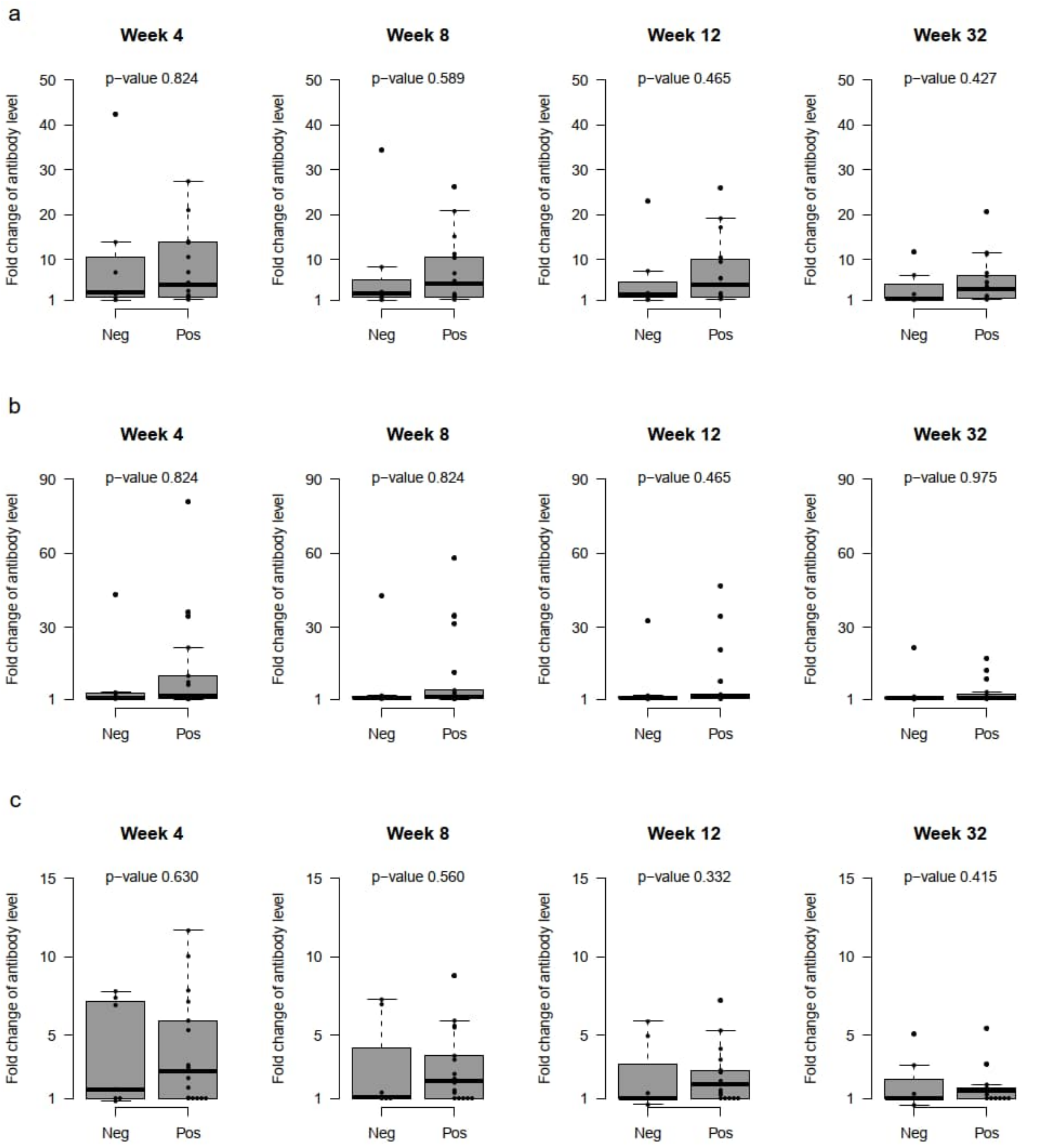

ESM Fig. 4. Fold-change (±SD) in IgG (a), IgM (b) and IgA (c) class antibody levels against Coxsackie B virus antigen in ELISA at weeks 4, 8, 12 and 32 (EOS visit) compared to baseline (week 0) in subjects who received either high or low dose of PRV-101. Responses are categorised based on the presence of neutralising antibodies against CVBs at base line. Neg = subjects who were negative for neutralising antibodies against all tested CVB serotypes (N=7). Pos = subjects who had neutralising antibodies against at least one CVB serotype (N=17). P-values represent statistical significance in Kruskal-Wallis rank sum test (R version 4.3.1). In the boxplot charts, the black line represents the median, the box spans from the first quartile (Q1) to the third quartile (Q3), and the whiskers are extending up to 1.5 times the IQ range.
